# Preoperative Oral Feeding in Infants with Congenital Heart Disease Within the First Month of Life is Associated with a Higher Likelihood of Freedom From Tube Feeding at Time of Postoperative Discharge

**DOI:** 10.1007/s00246-024-03750-z

**Published:** 2025-01-08

**Authors:** Aseel Dabbagh, Sarah Miller, Michael McCulloch, Geoffrey Rosenthal, Mark Conaway, Shelby White

**Affiliations:** 1 Division of Pediatric Cardiology, Department of Pediatrics, University of Virginia, Charlottesville, USA; 2 Division of Neonatology, Department of Pediatrics, University of Virginia, Charlottesville, USA; 3 Division of Translational Research and Applied Statistics, Department of Public Health Sciences, University of Virginia, Charlottesville, VA USA

**Keywords:** Congenital heart disease, Feeding tube, Neonates, Oral feeding

## Abstract

Though several studies have demonstrated that preoperative oral feeding (PO) can be safe in patients with congenital heart disease, they are commonly prohibited from doing so, potentially precluding the development of such skills. We sought to determine whether preoperative oral feeding is associated with freedom from tube feeding at postoperative discharge. Single-center, observational study including patients in the first month of life (≤ 30 days of age) who underwent a single cardiac surgery between 7/1/2017–6/30/2022 and survived to discharge. Preoperative PO was defined as any oral intake up to the day of cardiac surgery. General demographics and peri-operative characteristics were analyzed. A total of 235 patients were included of which 178 (78%) PO fed preoperatively, and 171 (73%) were discharged taking full PO. Those discharged without tube feeds received more preoperative oral feeds, were less likely to have a chromosomal abnormality/genetic syndrome, had lower STAT categories, and were less likely to have postoperative complications, vocal cord dysfunction or prolonged mechanical ventilation. Multivariate analysis found that any preoperative PO [odds ratio (OR) of 2.78 (CI 1.48, 5.24, p-value = 0.002)] and increasing amounts of PO were predictive of full PO intake at discharge [≤ 20 ml/kg/day (OR 2.06, CI 1.03, 4.14, *p*-value = 0.042) and > 20 ml/kg/day (OR 4.07, CI 1.88, 8.84, *p*-value = 0.004)]. Preoperative oral feeding is a strong predictor of discharging with full PO intake after cardiac surgery in the first month of life and that it may also improve with increasing volumes. Multi-institutional analyses are warranted.

**Supplementary Information:**

The online version contains supplementary material available at 10.1007/s00246-024-03750-z.

## Introduction

Approximately 40,000 children undergo congenital cardiac surgery annually, and with survival rates exceeding 95%, clinicians need to focus on optimizing neurodevelopmental outcomes. Decades of data have demonstrated that patients requiring surgery for congenital heart disease (CHD) experience a spectrum of neurologic (i.e., abnormal brain morphology/functional connectivity), intellectual (i.e., lower IQ), psychosocial (i.e., executive functioning and attention deficits) and developmental (oral-motor dysfunction, language delays) challenges [1]. One of the earliest and most significant such challenge is oral-motor dysfunction, present in about 22–48% of patients [2–5].

Oral feeding skills start to develop immediately after birth [6], but some newborns with CHD are prohibited from doing so in the setting of positive pressure ventilation, orofacial or gastrointestinal defects (i.e., tracheoesophageal fistula, duodenal atresia), neurologic impairment (i.e., hypotonia, hypoxic-ischemic encephalopathy, stroke) or other clinical factors (i.e., abnormal labs, vasoactive support requirement). In the absence of these limitations, however, some neonates with CHD are prevented from oral feeding due to the perceived risk of intestinal ischemia/necrotizing enterocolitis (NEC). Some are also provided the opportunity to oral feed but lack feeding cues. Regardless of the reason, those infants lose early opportunities to develop oral feeding skills instead attempting for the first time after cardiopulmonary bypass (CPB), anesthetics and neurosedatives, and ultimately require temporary or surgically placed feeding tubes, with a higher incidence in neonates [7]. Given this, we hypothesized that preoperative oral feeding would decrease the incidence of feeding tube requirement at the time of discharge after cardiac surgery in the first month of life.

## Materials and Methods

This was a single center, observational study performed at the University of Virginia (UVA) Children’s Hospital. Our institutional congenital cardiac surgical database was queried for infants ≤ 30 days of age at the time of surgical intervention between 7/1/2017–6/30/2022. Patients who underwent more than one congenital cardiac surgical intervention during initial hospitalization and those who died prior to discharge were excluded as outliers for their significantly longer postoperative lengths of stay. The center’s congenital cardiac surgical database query yielded data points which are standardly reported to the STS (The Society of Thoracic Surgeons) Congenital Heart Surgery Database [8]. The electronic medical record was used to gather additional and missing data points, including classification of single vs. biventricular CHD, ductal-dependent CHD, the presence of orofacial defects, whether the patient orally fed preoperatively and how much, route of intubation (oral vs. nasal vs. tracheostomy), and discharge route(s) for nutrition (full oral (PO) intake, oral plus nasogastric (NG) feeding tube (PO/NG), NG tube, and gastrostomy tube (GT)).

The amount of preoperative oral feeding was classified into three categories: those who did not feed, those who took trophic amounts, defined as ≤ 20 ml/kg/day up to the day of surgery, and those who took more than trophic amount, defined as > 20 ml/kg/day. Surgical procedures were classified as including any arch intervention or no arch intervention due to increased risk of vocal cord paresis associated with arch intervention [9].

All preoperative factors and postoperative complications were initially tabulated. Preoperative factors and postoperative complications were categorized per the STS Congenital Heart Surgery Database [8]. Preoperative factors and postoperative complications included are referenced in Table 1. Postoperative NEC data were obtained via chart review. Due to the low number of occurrences for some factors and complications, only the clinically relevant ones which occurred 10 or more times were included in analysis.

**Table 1:** Preoperative factors and postoperative complications tabulated

Preoperative factors	Postoperative complications
None	None
Non-invasive respiratory support requirement	Vocal cord dysfunction (diagnosed via laryngoscopy)
Invasive respiratory support requirement	Chylothorax
Cardiogenic shock	Sternum left open at end of surgical procedure
NEC* treated medically or surgically (*defined as outlined in Abbo, et al. [10])	NEC treated medically or surgically
Neurological complication including neurologic deficit, seizure, stroke or intracranial hemorrhage (IVH)	Respiratory insufficiency requiring: mechanical ventilatory support > 7 days, reintubation, or tracheostomy
Sepsis	Paralyzed diaphragm
Renal dysfunction	Pneumothorax requiring drainage
Colostomy requirement	Pleural effusion requiring drainage
Single lung	Unplanned non-cardiac reoperation during the postoperative time period
Steroid requirement for any reason	Unplanned cardiac reoperation during the postoperative time period
Coagulation disorder	Mechanical circulatory support requirement
Other	Unplanned interventional catheterization procedure
	Pericardial effusion requiring drainage
	Low cardiac output
	Unexpected cardiac arrest
	Stroke
	Seizure
	Arrhythmia requiring: drug therapy, temporary pacing, or permanent pacemaker placement
	Venous or intracardiac thrombus
	Wound dehiscence or infection including mediastinitis or sternal instability
	Sepsis
	Unplanned readmission within 30 days of surgery
	Other

## Statistical Methods

Pre- and postoperative characteristics were summarized by the preoperative PO intake amount and whether or not the patient was discharged taking full PO feeds. Categorical variables were summarized by proportions and compared using the chi-squared test for trend. Continuous variables were summarized by either the mean or standard deviation or by the median and quartiles and compared using the nonparametric Wilcoxon rank test. Variables that were clinically relevant and significantly associated with full PO intake on discharge were included as variables in a multivariable logistic regression model. Estimates and 95% confidence intervals in the logistic regression model were computed using the Firth penalized likelihood method [11]. All analyses were conducted in SAS 9.4 and GAUSS 24.0.

## Results

**Fig. 1:**
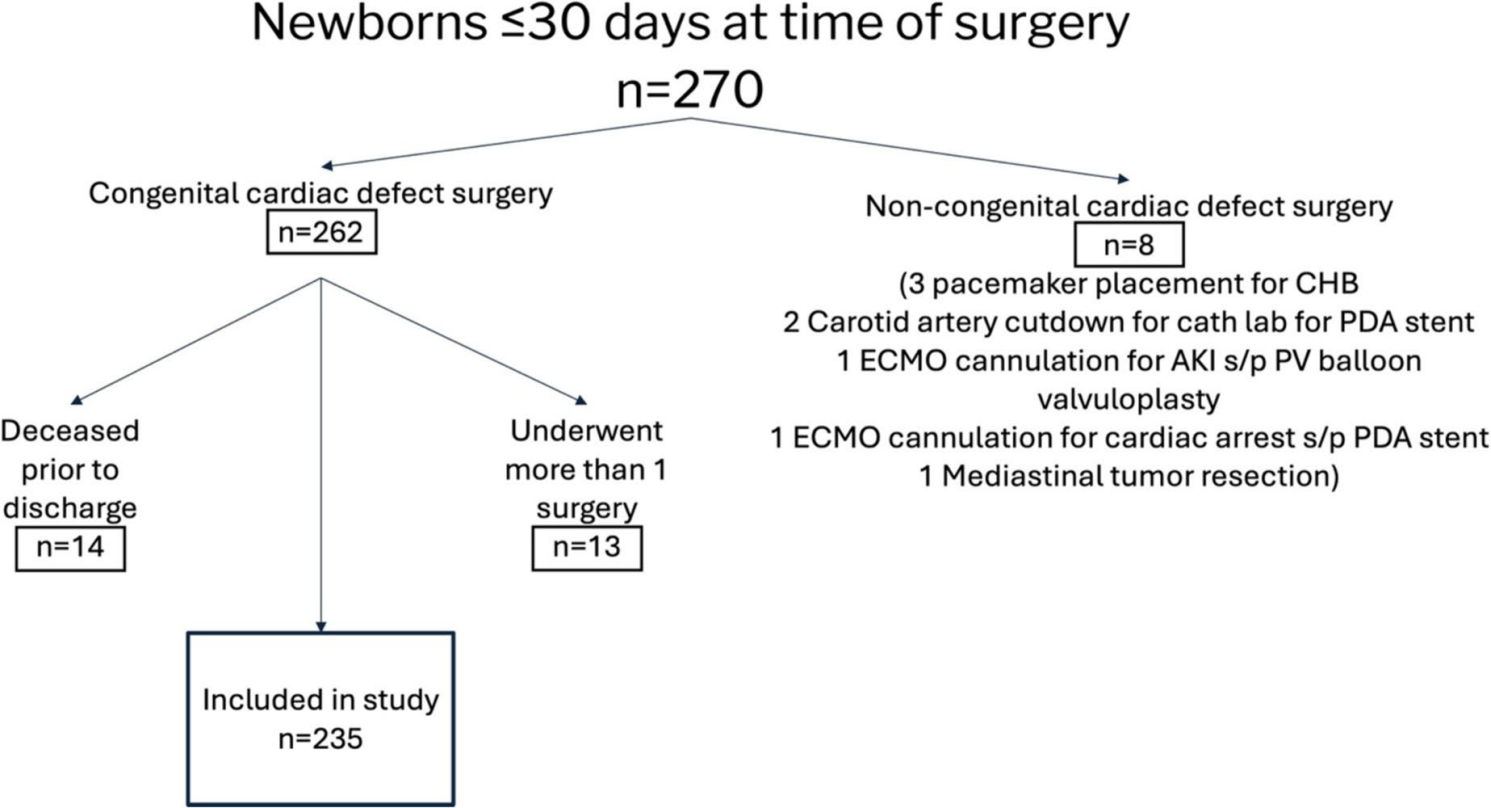
Patients included in study

The initial query resulted in 270 patients ≤ 30 days of age of which 262 underwent congenital cardiac defect surgery. Figure 1 demonstrates the breakdown of the final patient population.

Of the 235 patients, 178 (76%) fed orally in the preoperative period. At the time of discharge, 171 patients (73%) were discharged tolerating full PO intake; 28 (12%) had received a GT, 10 (4%) were receiving NG feeds, and 26 (11%) were receiving a combination of PO and NG feeds. There were no patients discharged tolerating any amount of oral feeds with a GT in place.

Table 2 demonstrates baseline demographic characteristics for those who were and were not fed preoperatively. Of those who were ductal-dependent for systemic circulation who fed preoperatively, 48 patients (33%) fed > 20 ml/kg/day. This was similar for those ductal-dependent for pulmonary circulation (*n* = 17, 35%). Overall, the groups did not differ significantly with the exception of age at surgery, need for invasive mechanical ventilation and number of preoperative factors. The predominant preoperative factors noted were non-invasive respiratory support to treat cardiorespiratory failure and invasive mechanical ventilation to treat cardiorespiratory failure. Only two patients developed NEC preoperatively (hypoplastic aortic arch on PGE, D-transposition of the great arteries with ventricular septal defect on PGE), one of which required surgical treatment. Gestational age was statistically but not clinically different between the groups.

**Table 2:** Demographics and peri-operative characteristics by preoperative oral feeding amount

Preoperative variable		Preoperative PO intake amount		
Total (n, %)		No (57)	Yes (178, 76%)	p-value
Sex	Male (140, 60%^a^)	33	107 (76%^b^)	0.910
	Female	23	72 (76%)	
Race	Caucasian (152, 65%)	34	118 (78%)	0.408
	African American	16	30 (65%)	
	Asian	2	6 (75%)	
	More than one race	1	7 (88%)	
	Unknown	4	17 (81%)	
Gest. Age, wks	Mean (SD)	38 (1.6)	38.5 (1.25)	0.014
Birth Wt, kg	Mean (SD)	3.15 (0.59)	3.2 (0.51)	0.284
Age at Surgery, d	Mean (SD)	7.81 (5.73)	10.1 (6.40)	< 0.001
Chrom. Abnorm./ Syndrome	No (192, 82%)	44	148 (77%)	0.312
	Yes	13	30 (70%)	
Orofacial Defects	No (226, 96%)	54	172 (76%)	0.517
	Yes	3	6 (67%)	
Diagnosis	Hypoplastic Aortic Arch (68, 28%)	12	56 (82%)	0.122
	Single-Ventricle CHD (72, 31%)	21	51 (71%)	
	Conotruncal CHD (72, 31%)	15	57 (79%)	
	Other CHD (23, 10%)	9	14 (61%)	
Ventricle	Bi (154, 66%)	33	121 (79%)	0.163
	Single	24	57 (70%)	
Ductal Dependent	Yes, Systemic (145, 62%)	38	107 (74%)	0.277
	Yes, Pulmonary (48, 20%)	12	36 (75%)	
	No (42, 18%)	7	35 (83%)	
Preoperative Factors	None (136, 58%)	25	111 (82%)	0.014
	Some	32	67 (68%)	
Non-Invasive Respiratory Support to Treat Cardiorespiratory Failure	No (222, 94%)	54	168 (76%)	0.919
	Yes	3	10 (77%)	
Invasive Mech. Vent. to Treat Cardiorespiratory Failure	No (157, 67%)	27	130 (83%)	< 0.001
	Yes	30	48 (62%)	

*PO* oral, *SD* standard deviation, *CHD* congenital heart disease, *Gest* gestational, *wks* weeks, *Wt* weight, *kg* kilograms, *d* days, *Chrom* chromosomal, *Abnorm* abnormality, *Mech* mechanical, *Vent* ventilation

^a^Percentage of patients within each category of variable (i.e., 60% male patients)

^b^Row percentage

Table 3 demonstrates the peri- and postoperative characteristics classified by whether or not patients were receiving full PO at the time of discharge. Those requiring complete or supplemental tube feeds at discharge had higher STAT (The Society of Thoracic Surgeons-European Association for Cardio-Thoracic Surgery) categories, endured more postoperative complications, were more likely to have vocal cord dysfunction and require prolonged mechanical ventilation. They also had significantly longer hospital and ICU lengths of stay (shown in Supplemental Table 1). Additionally, those receiving full PO feeds at discharge were more likely to be male (*p* = 0.002) and less likely to have a chromosomal abnormality (*p* = 0.002). There was no difference in those who had ductal-dependent circulation.

**Table 3:** Demographics and peri-operative characteristics by discharge feeding group

Variable		Discharged taking full PO		
Total (n, %)		No (64)	Yes (171, 73%)	p-value
Surgery Type	CPB (208, 88%^a^)	58	150 (72%^b^)	0.534
	No CPB	6	21 (78%)	
STAT Category	1–3 (146, 62%)	32	114 (78%)	0.019
	4–5	32	57 (64%)	
Surgical Procedure	Arch (187, 80%)	51	136 (73%)	0.979
	Other	13	35 (73%)	
Route Intubated	Oral (148, 63%)	42	106 (72%)	0.211
	Nasal	21	65 (76%)	
	Tracheostomy	1	0 (0%)	
Postoperative Complications	None (*n* = 91, 39%)	10	81 (89%)	< 0.001
	Some	54	90 (63%)	
Vocal Cord Dysfunction	No (215, 91%)	53	162 (75%)	0.004
	Yes	11	9 (45%)	
Chylothorax	No (223, 95%)	60	163 (73%)	0.626
	Yes	4	8 (67%)	
NEC	No (220, 94%)	58	162 (74%)	0.251
	Yes	6	9 (60%)	
Postoperative/Postprocedural Respiratory Insufficiency Requiring Mech. Vent. > 7 Days	No (197, 84%)	45	152 (89%)	0.001
	Yes	19	19 (50%)	

*PO* oral feeds, *CPB* cardiopulmonary bypass, *STAT* The Society of Thoracic Surgeons-European Association for Cardio-Thoracic Surgery, *NEC* necrotizing enterocolitis, *Mech* mechanical, *Vent* ventilation

^a^Percentage of patients within each category of variable (i.e., 88% of patients required CPB)

^b^Row percentage

## Modeling Results

A bivariate comparison between preoperative feeding and discharge taking full oral feeds was performed (Table 4). Compared to those who did not feed preoperatively, patients who fed any amount preoperatively were more likely to be discharged taking full oral feeds. On further analysis there was a notable dose–response relationship (Table 5), such that as volume of preoperative feeding increased, the proportion of patients discharged with full oral feeds also increased.

**Table 4:** Bivariate association of preoperative oral feeding and discharge taking full PO

		Discharged taking full PO		
		No (64)	Yes (171, 73%)	p-value
Preoperative Oral Feeding (n, %)	No (57, 24%^a^)	25	32 (56%^b^)	0.001
	Yes	39	139 (78%)	

*PO* oral feeds

^a^Percentage of patients within each category of variable (i.e., 24% patients did not feed orally preoperatively)

^b^Row percentage

**Table 5:** Bivariate association of preoperative oral feeding amount and discharge taking full PO

		Discharged taking full PO		
		No (64)	Yes (171, 73%)	p-value
Preoperative oral feeding (n, %)	No (57, 24%^a^)	25	32 (56%^b^)	0.001
	Yes, ≤ 20 ml/kg/d (91, 39%)	25	66 (73%)	
	Yes, > 20 ml/kg/d (87, 37%)	14	73 (84%)	

*PO* oral feeds, *ml* milliliter, *kg* kilograms, *d* day

^a^Percentage of patients within each category of variable (i.e., 24% patients did not feed orally preoperatively)

^b^Row percentage

Table 6 demonstrates an unadjusted logistic regression analysis of the effect of preoperative oral feeding on the probability of being discharged tolerating full oral feeds. Any volume of preoperative oral feeds significantly increased the likelihood of full oral feeds at discharge, but sub-analyses demonstrated step-wise increase in this probability when comparing trophic versus > 20 ml/kg/day feeds.

**Table 6:** Unadjusted logistic regression analysis of preoperative oral feeding and amount of preoperative oral feeding with discharge taking full oral feeds

Preoperative oral feeding comparison	Odds Ratio	95% CI	p-value
Yes vs No	2.78	1.48, 5.24	0.002
Yes, ≤ 20 ml/kg/d vs No	2.06	1.03, 4.14	0.042
Yes, > 20 ml/kg/d vs No	4.07	1.88, 8.84	0.004

*CI* confidence interval, *ml* milliliter, *kg* kilograms, *d* day

Multivariate logistic regression analysis using statistically significant demographics and peri-operative factors again demonstrated that any feeding and increase in volume of preoperative oral feeding was associated with a higher probability of discharge without the need for a feeding tube (Table 7). Having any postoperative complications decreased this probability.

**Table 7:** Multivariate logistic regression analysis of peri-operative characteristics found to be significantly associated with discharge taking full oral feeds

Variable	Comparison	Odds ratio	95% CI	p-value
Preoperative Oral Feeding	≤ 20 ml/kg/day vs No	2.25	1.06, 4.79	0.036
	> 20 ml/kg/day vs No	2.92	1.28, 6.69	0.011
Chrom. Abnormality	Yes vs No	0.45	0.23,1.06	0.070
STAT Category	4–5 vs 1–3	0.64	0.32,1.25	0.187
Postoperative Complication	Yes vs No	0.34	0.15,0.75	0.008
Vocal Cord Dysfunction	Yes vs No	0.38	0.14,1.05	0.061
Resp. Insufficiency > 7 Days	Yes vs No	0.60	0.26,1.38	0.230

*CI* confidence interval, *ml* milliliter, *kg* kilograms, *d* day, *Chrom* chromosomal, *STAT* The Society of Thoracic Surgeons-European Association for Cardio-Thoracic Surgery, *Resp* respiratory

## Discussion

With infants’ feeding skills beginning to develop at birth, it is important that they are provided the opportunity to work on these skills when clinically appropriate, thus decreasing the likelihood of developing oral aversion and the need for a feeding tube. Research has demonstrated many risk factors for oral aversion and feeding tube requirement in infants with CHD. Patients with longer nil per os (NPO) times, longer duration prior to oral feeding postoperatively, longer ICU LOS, and longer duration of mechanical ventilation were at increased likelihood of oral aversion that is significant at one year of age [12]. Our study demonstrated similar findings with increased likelihood of requiring tube feeds with no preoperative oral feeding and with longer duration of mechanical ventilation. Yildirim et al. demonstrated better odds of tube-free discharge and presumed decreased risk of oral aversion with nasotracheal intubation at the time of surgery [13]. However, in our study, there was no significant difference in those who achieved tube-free feeding at discharge based on intubation route. Other risk factors for discharging with a feeding tube included patient age, STAT score, and procedure group (specifically, aortic arch) [14]. We did find that patients who underwent higher STAT score procedures had a higher likelihood of discharging with tube feedings, however, there was no difference based on patient age (Supplemental Table 1) or procedure group. Our study overall demonstrated similar risk factors for discharging with a feeding tube.

Necrotizing enterocolitis is also a risk that has caused hesitancy among centers to enterally feed in the preoperative period [15]. This is due to the fear of further elevating this risk, especially in those with ductal-dependent systemic blood flow. The incidence of NEC in patients with congenital heart disease is known to be higher than the general population [16]. However, it has since been demonstrated that when patients are appropriately selected, preoperative enteral feeding is not associated with an increased incidence of NEC [17, 18]. Trophic feeds allow for intestinal mucosal development and administering them in the preoperative period leads to less fluid overload in the postoperative period, shorter postoperative intubation time, and faster time to reaching and tolerating full enteral feeding by tube and by mouth [19]. In our study, we again demonstrate no increase in the incidence of preoperative NEC despite the majority of our patients receiving some amount of preoperative feeds.

There is ample literature on the risks associated with CHD and feeding tube requirement, however, there is paucity of literature on the effect of preoperative oral feeding. The only data available for preoperative oral feeding and its association with postoperative oral feeding was shown in an analysis of the National Pediatric Cardiology-Quality Improvement Collaborative (NPC-QIC) dataset to assess preoperative feeding in general (oral or tube feeding), and it demonstrated that those patients that orally fed preoperatively did not have a significantly increased odds ratio of achieving tube-free feeding by 1 year of age [18]. The results of our study revealed a strong association with preoperative oral feeding and tube-free feeding at discharge. Even when adjusting for significant peri-operative characteristics, there is still a significantly higher odds of discharging taking full oral feeds. This NPC-QIC dataset analyzed only single ventricle patients. In our study, we analyzed all comers and showed there was no significant risk difference in achieving full oral feeds at discharge between single-ventricle and two-ventricle patients. Further analysis is required to compare odds of tube-free feeding by 1 year of age in our cohort.

## Conclusion

Preoperative oral feeding is a positive predictor of discharging with full PO intake after cardiac surgery. Chromosomal abnormalities/genetic syndromes, STAT category, having any postoperative complications, postoperative vocal cord dysfunction, and postoperative respiratory insufficiency requiring mechanical ventilation for more than 7 days were negative predictors. Even with adjustment for these factors, preoperative oral feeding is still a significant positive predictor of discharging with full PO intake with a notable response to increased volume of feeding. Allowing patients to practice their oral skills as much as clinically appropriate provides a significant advantage to newborn infants undergoing congenital cardiac surgery.

## Limitations

This is a single center retrospective observational study. The number of patients is also relatively small. Given that most of our data were obtained from our database, we must assume that the data were entered correctly. At our institution, we do not have a set protocol for feeding our patients preoperatively. Thus, there is selection bias as the decision to orally feed was often based on physician judgement. We also do not assess all of our patients for vocal cord dysfunction with laryngoscopy, so the number of patients who had this complication may be higher. However, even if the number of patients with vocal cord dysfunction were higher, it did not seem to preclude most patients (75%) classified as not having postoperative vocal cord dysfunction from going home with full oral feeds.

## Supplementary Information

Below is the link to the electronic supplementary material.

Supplementary file1 (DOCX 20 KB)

## Data Availability

No datasets were generated or analysed during the current study.
