# Supplementary material for: Preoperative Oral Feeding in Infants with Congenital Heart Disease Within the First Month of Life is Associated with a Higher Likelihood of Freedom From Tube Feeding at Time of Postoperative Discharge
Source: Pediatr Cardiol. 2025 Jan 8;47(1):221–8. doi: 10.1007/s00246-024-03750-z (PMC12827393; doi:10.1007/s00246-024-03750-z)
Supplement: Supplementary file 1 — Supplementary file1 (DOCX 20 KB) [file 246_2024_3750_MOESM1_ESM.docx]

**Supplemental Material**

| **Supplemental Table 1. Demographics and peri-operative characteristics by discharge feeding group.** | | | | |
| --- | --- | --- | --- | --- |
| Variable | | Discharge taking full PO | | |
| Total | | No (n = 64) | Yes (n = 171) | p-value |
| Sex (n, %) | Male (140, 60%^a^) | 28 | 112 (80%^b^) | 0.002 |
|  | Female | 36 | 58 (62%) |  |
| Race | Caucasian (152, 65%) | 36 | 116 (76%) | 0.459 |
|  | African American (46, 20%) | 17 | 29 (63%) |  |
|  | Asian (8, 3%) | 3 | 5 (63%) |  |
|  | More than one race (8, 3%) | 2 | 6 (75%) |  |
|  | Unknown (21, 9%) | 6 | 15 (71%) |  |
| Gestational Age, wks | Mean (SD) | 38.2 (1.4) | 38.5 (1.4) | 0.085 |
| Birth Wt, kg | Mean (SD) | 3.14 (0.51) | 3.25 (0.54) | 0.329 |
| Age at surgery, days | Mean (SD) | 11.2 (7.7) | 9.3 (5.2) | 0.407 |
| Chromosomal Abnormality/Syndrome | No (192, 82%) | 44 | 148 (77%) | 0.002 |
|  | Yes | 20 | 23 (53%) |  |
| Orofacial Defects | No (226, 96%) | 61 | 165 (73%) | 0.675 |
|  | Yes | 3 | 6 (67%) |  |
| Diagnosis | Hypoplastic Aortic Arch (68, 29%) | 19 | 49 (72%) | 0.297 |
|  | Single Ventricle CHD (72, 31%) | 24 | 48 (67%) |  |
|  | Conotruncal CHD (72, 31%) | 14 | 58 (81%) |  |
|  | Other (23, 9%) | 7 | 16 (70%) |  |
| Ventricle | Bi (154, 66%) | 37 | 117 (76%) | 0.128 |
|  | Single | 27 | 54 (67%) |  |
| Ductal dependent | Systemic (145) | 45 | 100 (69%) | 0.151 |
|  | Pulmonary (48) | 8 | 40 (83%) |  |
|  | No (42) | 11 | 31 (74%) |  |
| Preoperative Factors | None (136, 58%) | 36 | 100 (74%) | 0.758 |
|  | Some | 28 | 71 (71%) |  |
| Preoperative Non-Invasive Respiratory Support to Treat Cardiorespiratory Failure | No (222, 94%) | 60 | 162 (73%) | 0.768 |
|  | Yes | 4 | 9 (69%) |  |
| Preoperative Invasive Mech. Ventilation to Treat Cardiorespiratory Failure | No (157, 67%) | 39 | 118 (75%) | 0.242 |
|  | Yes | 25 | 53 (68%) |  |
| Total CPB Time (min) | Mean (SD) | 148 (66) | 152 (72) | 0.595 |
| Total CC Time (min) | Mean (SD) | 74 (45) | 86 (57) | 0.177 |
| ICU LOS (days) | Median (Q1-Q3) | 25 (9-37) | 8 (4-13) | <0.001 |
| Hospital LOS (days) | Median (Q1-Q3) | 45 (31-56) | 26 (20-34) | <0.001 |
| Abbreviations: PO, oral feeds; wks, weeks; SD, standard deviation; kg, kilograms; CHD, congenital heart disease; Mech, mechanical; CPB, cardiopulmonary bypass; min, minutes; CC, cross clamp; LOS, length of stay; Q, quartile.  ^a^ Percentage of patients within each category of variable (i.e., 60% male patients).  ^b^ Row percentage. | | | | |
